# A randomised controlled trial investigating the effect of n-3 long-chain polyunsaturated fatty acid supplementation on cognitive and retinal function in cognitively healthy older people: the Older People And n-3 Long-chain polyunsaturated fatty acids (OPAL) study protocol [ISRCTN72331636]

**DOI:** 10.1186/1475-2891-5-20

**Published:** 2006-08-31

**Authors:** Alan D Dangour, Felicity Clemens, Diana Elbourne, Nicky Fasey, Astrid E Fletcher, Pollyanna Hardy, Graham E Holder, Felicia A Huppert, Rosemary Knight, Louise Letley, Marcus Richards, Ann Truesdale, Madge Vickers, Ricardo Uauy

**Affiliations:** 1 Nutrition and Public Health Intervention Research Unit, Department of Epidemiology and Population Health, London School of Hygiene & Tropical Medicine, London, UK; 2 Medical Statistics Unit, Department of Epidemiology and Population Health, London School of Hygiene & Tropical Medicine, London, UK; 3 Medical Research Council General Practice Research Framework, London, UK; 4 Non-Communicable Disease Epidemiology Unit, Department of Epidemiology and Population Health, London School of Hygiene & Tropical Medicine, London, UK; 5 Clinical Epidemiology and Biostatistics Unit, Royal Children's Hospital, Melbourne, Australia; 6 Department of Electrophysiology, Moorfields Eye Hospital, London, UK; 7 Department of Psychiatry, University of Cambridge, Cambridge, UK; 8 Department of Epidemiology and Public Health, University College London, London, UK; 9 Centre for Research in Primary and Community Care, University of Hertfordshire, Hatfield, UK

## Abstract

The number of individuals with age-related cognitive impairment is rising dramatically in the UK and globally. There is considerable interest in the general hypothesis that improving the diet of older people may slow the progression of cognitive decline. To date, there has been little attention given to the possible protective role of n-3 long-chain polyunsaturated fatty acids (n-3 LCPs) most commonly found in oily fish, in age-related loss of cognitive function. The main research hypothesis of this study is that an increased dietary intake of n-3 LCPs will have a positive effect on cognitive performance in older people in the UK.

To test this hypothesis, a double-blind randomised placebo-controlled trial will be carried out among adults aged 70–79 years in which the intervention arm will receive daily capsules containing n-3 LCP (0.5 g/day docosahexaenoic acid and 0.2 g/day eicosapentaenoic acid) while the placebo arm will receive daily capsules containing olive oil. The main outcome variable assessed at 24 months will be cognitive performance and a second major outcome variable will be retinal function. Retinal function tests are included as the retina is a specifically differentiated neural tissue and therefore represents an accessible window into the functioning of the brain.

The overall purpose of this public-health research is to help define a simple and effective dietary intervention aimed at maintaining cognitive and retinal function in later life. This will be the first trial of its kind aiming to slow the decline of cognitive and retinal function in older people by increasing daily dietary intake of n-3 LCPs. The link between cognitive ability, visual function and quality of life among older people suggests that this novel line of research may have considerable public health importance.

## Background and rationale

The number of individuals with age-related cognitive impairment is rising dramatically in the UK [1] and globally [2]. Global burden of disease estimates now place dementia above stroke, musculoskeletal disorders, cardiovascular disease and all forms of cancer in terms of the percentage of years lived with disability in people aged 60 years and older [2]. Defining simple and effective strategies to prevent or delay cognitive impairment among older people is therefore a priority for healthcare and social services.

There has been specific interest in the hypothesis that enhancing the diet of older people may act to slow the progression of cognitive decline. The importance of good nutrition among older people for the maintenance of health has long been advocated, and evidence-based dietary recommendations for older people have been published [3]. However, for a variety of functional, physiological, psychological and social reasons older people are nutritionally vulnerable, and frequently consume diets that are poor in both quality and quantity resulting in macronutrient and micronutrient under-nutrition.

Interest has recently turned to the potential importance of n-3 long-chain polyunsaturated fatty acids (n-3 LCPs), largely obtained from oily fish, in the maintenance of good cognitive health. The brain is particularly rich in the n-3 LCP docosahexaenoic acid (DHA), and n-3 LCPs have repeatedly been shown to be crucial to brain development in humans [4]. Age-related decrease in n-3 LCP level in total brain lipids have been reported in humans, and it has been postulated that this decline is correlated in part with age-related deterioration of functions of the central nervous system [5,6]. This findings may be particularly relevant in the UK since recent survey data [7] demonstrates that older people in the UK habitually consume a diet that is low in fish.

A recent cross-sectional survey has reported that higher fatty fish and n-3 LCP consumption is associated with reduced risk of cognitive impairment [8], and prospective studies have demonstrated that increased fish consumption is associated with decreased risk of dementia [9,10] and Alzheimer's disease [11] among older people. Data from a cohort study recently suggested that reported daily use of fish-oil supplements was associated with improved scores in cognitive function at age 64 years, even after correcting for childhood cognitive ability [12].

Numerous randomised controlled trials have demonstrated that a relative sufficiency of n-3 LCPs results in improved sensitivity to light in infancy [4]. It is thought that this results from the ability of n-3 LCPs (specifically DHA) in rod outer segments of the retina, to enhance photoreceptor signal transduction processing. Relative inadequacy of DHA results in decreased signal transduction, and DHA deficient subjects therefore require greater light stimulation to elicit the same level of photoelectric response. In addition DHA plays a key role in photoreceptor growth and functional development, a role which has recently been demonstrated to be mediated by DHA effects on gene expression [13].

A highly significant change occurring in the ageing retina is a decrease in phototransduction efficiency in rods resulting in a decreased sensitivity of rods to light [14]. Part of this decreased sensitivity may be associated with reduction in DHA levels, such as also occurs in the ageing brain, and may be susceptible to dietary manipulation. It is hypothesised that increasing dietary DHA intake among older people will result in increased levels of DHA in the brain and retina, and therefore enhance phototransduction efficiency and consequently light sensitivity.

A recent Cochrane review [15] concluded that there was a growing body of evidence from biological, observational and epidemiological studies that suggested a protective effect of n-3 LCPs against dementia. However, the Cochrane review team was unable to locate a single published randomised controlled trial on which to base recommendations for the use of dietary or supplemental n-3 LCPs for the prevention of cognitive impairment or dementia. The current study will be the first trial of its kind aiming to slow the decline of cognitive and retinal function in older people by increasing daily dietary intake of n-3 LCPs. The link between cognitive ability, visual function and quality of life among older people suggests that this novel line of research may have considerable public health importance. Furthermore, improving the quality of life and independence of older people, may help to reduce the number of disability-adjusted life-years lost through poor cognitive and retinal function in the UK.

## Design and methodology

### Design

**Figure 1:**
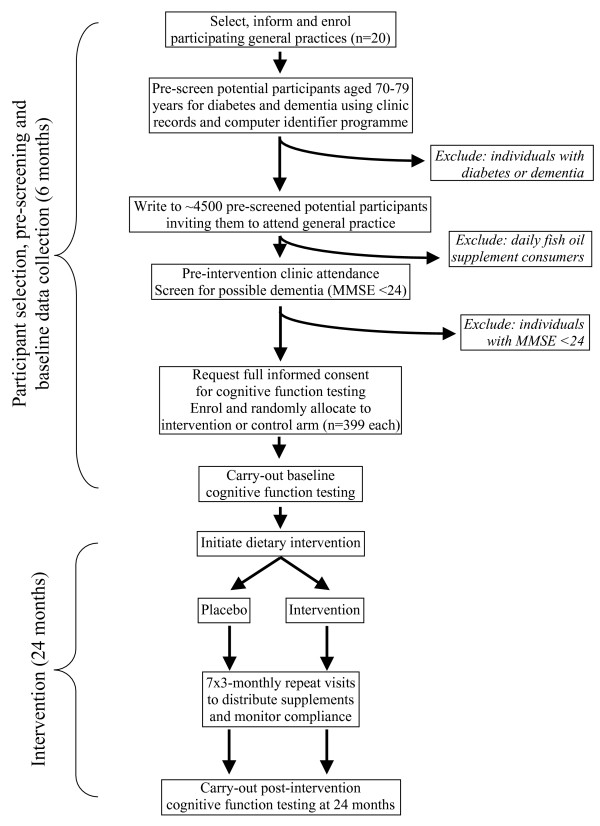
Flow-chart of cognitive function study protocol.

**Figure 2:**
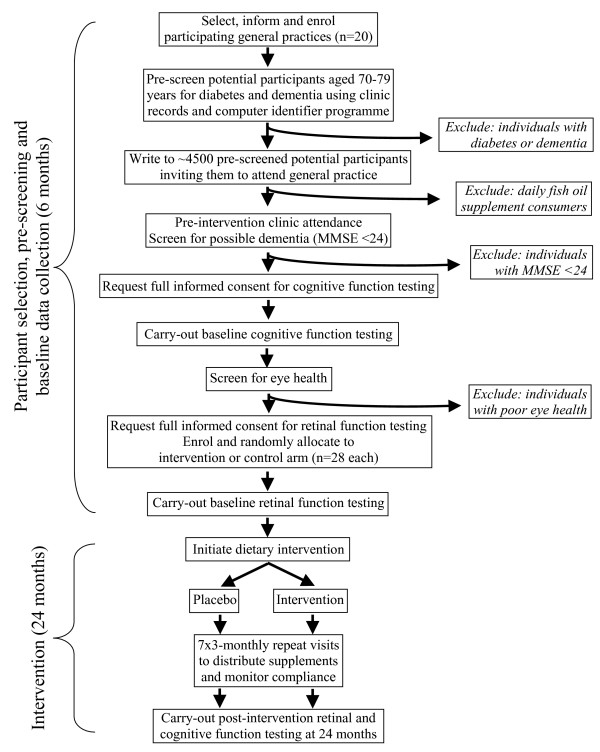
Flow-chart of retinal function study protocol.

The study is designed as a double-blind randomised placebo-controlled trial. The procedures are illustrated schematically in Figures 1 and 2, and detailed in the text. The trial aims to test two primary hypotheses.

### Study hypotheses

**Cognitive function study **– for healthy, cognitively normal adults aged 70–79 years of age, daily supplementation with n-3 LCPs (500 mg DHA and 200 mg EPA) will slow the rate of cognitive decline.

**Retinal function study **– for healthy, cognitively normal adults aged 70–79 years of age, daily supplementation with n-3 LCPs (500 mg DHA and 200 mg EPA) will improve visual function by enhancing rod photoreceptor response to light and visual-cortical integration.

### Inclusion criteria

#### (i) Centres

20 National Health Service general practices, members of the Medical Research Council General Practice Research Framework (GPRF) will be recruited. The retinal function sub-study practices will be situated in the Southeast of England to facilitate attendance at Moorfields Hospital.

#### (ii) Patients

Participating general practices, will draw a sample of healthy, cognitively-normal adults aged 70–79 years from their practice registers. These potential participants will be pre-screened for eligibility, by study nurses employed by each practice, using available clinic records and a GPRF computer identifier programme. Individuals currently diagnosed with either diabetes (Type I or Type II) or dementia will be excluded. The exclusion of individuals with diabetes is necessary as these individual will have raised susceptibility to vascular and neural damage and may therefore be less sensitive to the proposed intervention. Individuals with pre-diagnosed dementia will be excluded as the intervention is aimed at delaying cognitive decline in people who are currently not demented. The list of potential participants will be checked by the named General Practitioner in the clinic who will use discretion to exclude individuals not deemed suitable to take part in the study (e.g. recent bereavement, terminal illness).

Eligible individuals identified by this process will each receive a letter and information sheet from their general practice outlining the nature and importance of the study. The letter will also act as a screen for potential participants who are currently consuming fish-oil supplements. Participants reporting the current daily use of fish-oil supplements will be excluded from the trial. Potential participants will be invited to make an appointment with the research nurse at their local general practice. Participants will be offered the use of a taxi, or their travel costs on public transport or mileage in private cars to attend the appointment up to a maximum of £10. The invitation letter will explicitly state that if they do not wish to take part in the study, it will not prejudice the quality of health care provision from their general practice.

On attending their general practice, potential participants will be fully informed by the research nurse about the nature and relevance of the trial, and exactly what will be involved if they agree to take part. All potential participants will be told that a prerequisite of their joining the study will be that they agree not to initiate non-study fish-oil supplement use over the course of the trial. Potential participants who remain interested in taking part in the study, will then be asked to give consent to undergo a short cognitive screen, the Mini Mental State Examination (MMSE). The MMSE is an easy to administer test that has been widely validated as a screen for dementia and will be used to exclude participants with low cognitive status [16]. Participants with an MMSE score of less than 24 (out of a maximum of 30) will not be included in the trial, and will be offered a referral to their General Practitioner for further monitoring. The cut-off of less than 24 has traditionally been used as a marker for possible dementia in adults. It is possible that individuals with an MMSE of 24 or greater may have mild dementia. However, given the potential public health relevance of this trial, the purpose of the proposed cut-off is to exclude individuals with possible frank dementia rather than select a highly functioning group of individuals. Individuals with an MMSE score below 24 will be thanked for their time and co-operation.

Participants who score MMSE of 24 or greater will be invited to give full, informed written consent to take part in the main cognitive function study. Individuals unwilling to participate further will be thanked for their time and co-operation.

Individuals giving informed consent will be enrolled in the trial. The research nurse will telephone the central randomisation service to register the participant, giving identifying details and the participant's age. Randomisation will allow secure blind allocation of eligible people to one or other arm of the study. Following random allocation a study number will be given for each study participant. This will also be used to identify the supply of capsules to be prescribed for each participant. The study number will be entered on the participant's entry form. Pre-labelled identical-looking packs of capsules (see *Dietary Intervention *below) will be stored securely in the general practice. Minimisation criteria will be used to ensure a balance of key prognostic factors using the following two criteria: age group (70–74 and 75–79 years) and general practice.

Participants will then be assessed by completing questionnaires and undertaking a series of tests (see *Baseline Data Collection *below). The full set of baseline tests will take approximately 60 minutes to complete. Any couples recruited to the study will undertake their assessments separately. Participants will be provided with the contact details of the study nurses and trial manager. Information will also be available from the trial web-site [17] for those participants who wish to use this method of obtaining information. Participants will be thanked for their time and co-operation.

## Cognitive function study

### Outcome measures for cognitive function study

#### Primary outcome

Change in cognitive function at 24 months determined by the California Verbal Learning Test. This is a test of memory of 16 words.

#### Secondary outcomes

Cognitive performance as measured by immediate and delayed recall of a short story, tests of prospective memory, timed letter search/cancellation task, verbal fluency, digit span forwards and backwards, symbol digit modalities test, simple and choice reaction time, and spatial memory.

Psychological health.

Compliance determined by counting the number of capsules remaining every 3 months, and by measuring the change in n-3 LCP concentration in buccal epithelial cells over 24 months.

Blood pressure.

Change in Body Mass Index (a measure of body size).

Number of hospital admissions for cardiovascular events over 24 months.

Death.

### Sample size

The sample size required for this trial is based on a 0.3SD difference between trial arms in long-delay free recall of List A of the California Verbal Learning Test over the 24 months of intervention. To detect a 0.3SD difference between trial arms, with 90% power and 1% significance, 332 individuals are required per treatment group. Allowing for 20% drop-out over 24 months of intervention, the total sample size required for the study is 798 individuals. A difference of 0.3SD is clinically relevant and would be equivalent to individuals in the intervention arm being able to remember one more word out of the 16 in the California Verbal Learning Test than those in the placebo arm [18].

### Recruitment

In order to attain this sample size it is expected that a sample of approximately 4500 individuals, pre-screened for diabetes and dementia, will initially be drawn from the registers of 20 participating general practices (around 225 patients aged 70–79 years per practice). A conservative estimate of the proportion of eligible individuals who will agree to participate in the trial is 20%. It is estimated that it will take 6 months to enrol the full sample for this trial.

### Baseline (pre-intervention) cognitive function data collection

#### At trial entry to enable randomisation

Initials

Date of birth

Gender

General practice number

MMSE score

Consent

### Baseline data collection

1. Psychological health: the 30–item General Health Questionnaire (GHQ-30) will be used to assess the affective state of participants [19]. The GHQ-30 is quick and easy to administer, and an assessment of mood is important as it may be associated with cognitive performance.

2. Educational achievement: the widely used Burnham Scale of educational achievement will be used [20]. This is an important covariate as educational level has been shown to be strongly related to cognitive performance.

3. Blood pressure: this is necessary as hypertension is an important consequence of the metabolic syndrome, and individuals with high blood pressure may have a raised susceptibility to vascular and neural damage. Baseline blood pressure will therefore be used as a covariate in analysis. Blood pressure will be measured using the automated DYNAMAP device. Two measurements each of systolic and diastolic pressure will be taken in the sitting position (the mean of the two measurements will be used in analysis); participants will have rested for 5 minutes before their blood pressure is measured and there will be a minimum of 5 minutes between the two measurements. Normal general practice procedures will be followed for the management and treatment of high blood pressure.

4. Cardiovascular health: any history of hospital admission for myocardial infarction (MI) or stroke over the preceding 5 years will be abstracted from patient notes.

5. National Health Service number: So that the study organisers do not lose contact with patients should they move address and also to follow up on health status, participants are being asked to give their agreement for their names and NHS numbers to be sent to the NHS Central Register.

6. Basic anthropometric measures: height and weight will be measured using standardised procedures in order to enable the calculation of the Body Mass Index: a basic marker of body size.

7. Swab of cheek cells: nurses will collect a sample of buccal mucosal epithelial cells from each participant. This is a relatively non-invasive procedure in which a sterile wooden spatula is scraped briskly along the inside of the cheek (about 10 strokes on each cheek). The cheek cells will be sent to laboratories, in sterile containers, to be analysed for their n-3 LCP concentration.

8. Fish consumption: two simple questions regarding habitual fish consumption will be asked. Firstly, a question regarding the frequency of fish consumption, and second, participants will be asked to list the three species of fish they consume most frequently.

9. Cognitive ability: the participants will be asked to complete a validated series of cognitive tests. It is currently not known which, if any, cognitive domain will be most affected by n-3 LCP supplementation and therefore a series of tests have been selected which cover the major domains (memory, attention, psychomotor speed and executive function):

a. Brief assessment of subjective symptoms of cognitive impairment (memory, language/word-finding, concentration).

b. Immediate and delayed recall of a short story (from the Wechsler Memory Scale).

c. Immediate and delayed recall of a 16-item word list (California Verbal Learning Test).

d. Three tests of prospective memory i.e. remembering to carry out instructions without being reminded.

e. Timed letter search/cancellation task (attention/psychomotor speed/executive function).

f. Verbal fluency – naming animals (word-finding/executive function).

g. Digit span forwards and backwards (working memory/executive function) from the Wechsler Adult Intelligence Scale.

h. Symbol digit modalities test (attention/psychomotor speed/executive function).

i. Simple (psychomotor speed) and choice (decision speed) reaction time.

j. Spatial memory (memory/visual spatial function).

### Dietary intervention

Following the completion of the series of tests detailed above, those participants not enrolled in the retinal function study will be introduced to the dietary intervention. The dietary intervention will be a daily dietary supplement in the form of capsules which will be identical in size, shape, colour and smell for both the intervention and placebo arms of the trial. Given the lack of trial evidence to support the use of any particular level of dietary supplementation, the dose selected in the OPAL study was based on the following considerations: the UK Food Standards Agency currently recommends that men and post-reproductive age women consume one to four, 140 g portions of oily fish a week [21]; typical dietary recommendations, such as those of the World Health Organisation fall in the range 0.3–0.5 g n-3 LCPs daily [22]; the dose of n-3 LCPs generally recognised as safe (GRAS) is 3 g per day.

Taking these considerations into account, a pragmatic decision was made on the dose for the OPAL study. The intervention arm will be asked to consume two 650 mg soft-gel capsules daily containing a total of 500 mg DHA and 200 mg EPA. This is equivalent to approximately 4.9 g of n-3 LCPs a week (0.7 g/day), a level that can be achieved via the consumption of approximately 300 g (slightly more than 2 portions) of fatty-fish a week. This level of supplementation falls well below the upper GRAS level and should thus be safe. The placebo arm will be asked to consume two identical 650 mg capsules containing olive oil daily, this oil is rich in n-9 fatty acids and will thus have a minimal effect on the 18:2 n-6/18:3 n-3 ratio. Changes in the n-6/n-3 ratio may affect prostanoid balance which affects vascular and inflammatory responses. The higher DHA to EPA ratio in the supplement is justified by the rationale of the study, which prioritises the neuroprotective effect of DHA over the vascular and anti-thrombotic effect of EPA.

The research nurses will explain the importance of consuming the capsules every day, suggesting that it should become part of their daily routine, for example by always consuming the capsules at breakfast-time. Participants will be given a 3-month supply of capsules at the baseline clinic visit, and asked to attend the clinic every 3-months throughout the 24-month course of the trial i.e. at 3, 6, 9, 12, 15, 18 and 21 months. Participants will be sent reminders to collect repeat supplies of capsules.

The participants enrolled in the retinal function study will be introduced to the dietary intervention after their baseline retinal function test (see below). These participants will be invited back to the general practice after retinal function testing to be given their dietary supplements.

### Data collection at 3, 6, 9, 12, 15, 18, and 21 months

Participants will have appointments to meet the research nurse every 3 months throughout the study (8 repeat visits in total including 24 month assessment). During these appointments, participants will be reminded of the importance of the study and of the need to comply with the study protocol. The research nurses will also record any information the participants volunteer regarding any discomfort caused by the capsules. The recognised side-effects of n-3 LCP capsules include belching, flatulence, abdominal discomfort and loose stools. These discomforts are generally mild and decrease over time, and the research nurses will provide reassurance to any concerned participants.

Participants will be asked to bring their dietary capsule containers with them to the meeting so that any remaining capsules can be counted as a measure of compliance. Buccal cell swabs will also be taken during these repeat appointments from a random 20% sample every 6 months (i.e. at 6, 12 and 18 months) as a further measure of compliance. Participant records from the general practice will be consulted every 3 months in order to record any hospital admissions for MI or stroke over the intervening 3 month period.

### Assessment at 24 months

A final evaluation of the cognitive function of all trial participants will be carried out after 24 months of intervention. Participants will be invited to attend their general practice and will be assessed for:

1. Psychological health: as at baseline.

2. Blood pressure: as at baseline.

3. Cardiovascular health: record of hospital admissions for MI or stroke between the 21 and 24 month appointments.

4. Basic anthropometric measures: as at baseline.

5. Buccal cell DHA concentration: as at baseline. Collected as a marker of the impact of the intervention on buccal cell DHA concentration, and compliance in the intervention arm of the trial.

6. Cognitive function: as at baseline.

Participants will be offered the use of a taxi, or their travel costs on public transport or mileage in private cars to attend the appointment up to a maximum of £10.

### Data analysis for assessing change in cognitive function

Primary analysis will be carried out based upon the groups as randomised ("intention to treat"). Results will be presented as appropriate effects sizes with a measure of precision (95% confidence intervals). Covariates such age, gender, and baseline blood pressure and DHA concentration will be adjusted for in the analysis. Further exploratory analyses will be based on those patients who fully follow the treatment protocol.

## Retinal function study

### Outcome measures for retinal function study

#### Primary outcome

Change in rod sensitivity over 24 months of intervention as measured by electroretinogram.

#### Secondary outcomes

Colour vision measured by detecting sensitivity to colour contrast which is a good marker of central retinal function.

Eye health assessed by carrying out a full ophthalmic examination.

### Sample size

The calculation for the sample size required to demonstrate a significant difference between the placebo and intervention arms of the trial is based on the decline in rod sensitivity with age [14]. In line with previous studies on the effect of DHA during infant development, we anticipate that an increase in dietary intake of DHA will significantly improve rod sensitivity; increases of the order of +2SD in rod sensitivity have been demonstrated in studies of DHA supplementation in preterm infants [23,24]. Considering the complexity of the study and the limited significance of small changes in retinal sensitivity we have defined a biologically significant effect as an increase of 1SD. To detect this effect with 90% power and 5% significance, the sample size required is 22 per group. Allowing for 25% drop-out during the trial, the total sample size required for this sub-study is 56 individuals.

### Recruitment

Following the initial selection of 20 general practices to be involved in the trial, 4–6 of these practices will be selected to take part in the retinal function study. Potential participants from these general practices will be informed about the retinal function study as well as the cognitive function study, and will be invited to take part in both studies.

During the initial meeting at the general practices, the research nurses will fully outline the nature of the trial to potential participants, and in addition, the nurses will describe the objectives and methods of the retinal function tests. Potential participants who remain interested in taking part in either the cognitive study or the cognitive and retinal function study, will then be asked to give consent to undergo a short cognitive screen, the MMSE (as outlined above). Participants who have an MMSE score of greater than or equal to 24 will be invited to give full, informed consent to take part in the cognitive function study. Individuals unwilling to participate further will be thanked for their time and co-operation.

Sufficient information about participants giving informed consent to take part in the cognitive function study will be transmitted to the central randomisation service to allow random allocation to one or other arm of the study. The allocation will be minimised by age group (70–74 and 75–79 years) and centre. Participants will then undergo the series of tests outlined above for the cognitive function study (see *Baseline cognitive function data collection*).

After the collection of baseline cognitive function data participants will be invited to consider the retinal function study. Participants will be free to opt in or out of the retinal function study. It will be explicitly stated that refusing to take part in the retinal function study will not prejudice the quality of treatment and support that they will receive in the cognitive function study or from the general practice. Participants expressing an interest in taking part in the retinal function study will be asked for consent to carry-out a simple visual test (the Logmar test) and respond to a few questions on eye health.

Any participant reporting a personal or family history of genetically-determined ocular or neurological disease, diabetes, glaucoma or age-related macular degeneration will be excluded from the retinal function study. Participants on the retinal function study will also be required to have a Logmar visual acuity of +0.2 or better. Participants reporting poor eye-health or/and those with visual acuity scores below +0.2 will be excluded from the retinal function study (but not the trial in general) and offered a referral to their General Practitioner for further monitoring.

Participants with good eye-health and scoring +0.2 or better on Logmar will be invited to give full, informed consent to take part in the retinal function study. Following the completion of the baseline cognitive function testing, participants will be given an appointment at Moorfields Eye Hospital for baseline retinal function testing. These participants will not receive the dietary intervention until they have completed their baseline retinal function tests.

### Baseline (pre-intervention) retinal function data collection

1. Full electroretinogram (ERG): the ERG is a mass electrical response of the retina to a luminance stimulus. It is recorded through an electrode placed on the surface of the eye, light stimuli are provided using a Ganzfeld dome stimulator. The response typically consists of an "a-wave" followed by a "b-wave". The first 10–12 milliseconds of the a-wave arises in relation to photoreceptor hyperpolarisation, and the slope of the linear portion of the a-wave can be related to the kinetics of phototransduction. The b-wave is generated in the inner nuclear layer of the retina, principally the ON-bipolar cells. Further recording will examine the a-wave evoked by a brighter flash specifically to examine the characteristics that can be related to phototransduction.

2. Colour vision: trial participants will be tested for colour vision which is a good measure of central retinal function. This will be performed using a computerised system that enables colour contrast sensitivity assessment by measuring thresholds in the protan and tritan colour confusion axes.

3. Ophthalmic examination: this will be conducted in order to determine the "health" of the eye at baseline. Participants will be fully informed of the health of their eyes. In addition, fundus photography will be performed such that if any changes in the ocular fundus occur that can be putatively related to the dietary supplementation, documentary evidence will be available.

It is anticipated that the duration of the full protocol for eye examination will be in the region of 2 hours. Although these examinations may cause slight discomfort to some participants, they are generally very well tolerated. ERG is a standard ophthalmological investigation, and Moorfields Eye Hospital performs approximately 2000 ERGs each year to standards in excess of those recommended by the International Society for Clinical Electrophysiology of Vision [25]. Participants will be offered the use of a taxi, or their travel costs on public transport or mileage in private cars to attend the baseline appointment at Moorfields Eye Hospital up to a maximum of £20.

### Dietary intervention

Following the completion of the cognitive function test and the retinal function tests, trial participants will be given an appointment to return to the general practice to be introduced to the dietary intervention (see *Dietary intervention *above).

### Data collection at 3, 6, 9, 12, 15, 18, and 21 months

Participants involved in the retinal function study as well as the cognitive function study will have appointments to meet the research nurse every 3 months throughout the study in an identical manner to those only involved in the cognitive function study (see above).

### Final (post-intervention) retinal function data collection

A final evaluation of the retinal function of trial participants will be carried out after 24 months of intervention. Study participants included in the retinal function study will be invited to attend Moorfields Eye Hospital where they will be assessed as follows:

1. Full electroretinogram: as at baseline.

2. Colour vision: as at baseline.

3. Ophthalmic examination: as at baseline.

Participants will be fully informed of the results of their eye examination, and offered the use of a taxi, or their travel costs on public transport or mileage in private cars to attend the 24 month appointment at Moorfields Eye Hospital up to a maximum of £20.

### Data analysis for assessing change in retinal function

Primary analysis will be carried out based upon the groups as randomised ("intention to treat"). We will test between group differences in change in rod sensitivity over the 24-month follow-up. We will carry out secondary outcome analysis on change in colour vision based on colour contrast specificity. Covariates such as age, gender, and baseline blood pressure and DHA concentration will be adjusted for in the analysis. Results will be presented as appropriate effects sizes with a measure of precision (95% confidence intervals). Further exploratory analyses will be based on patients fully following the treatment protocol.

## Trial organisation

### Investigators

1. *Alan Dangour *(Principal Investigator PI): public health nutritionist based at the London School of Hygiene & Tropical Medicine (LSHTM). Will co-ordinate and manage the overall running of the trial and will be closely involved in data analysis and paper writing.

2. *Ricardo Uauy*: senior public health nutritionist based at LSTHM.

3. *Astrid Fletcher*: senior epidemiologist based at LSHTM.

4. *Diana Elbourne*: senior statistician based at LSHTM. Has extensive RCT experience, will co-ordinate activity at the LSHTM Data Coordinating Centre (DCC) with particular responsibility for data management, entry and analysis.

5. *Ann Truesdale*: trial's advisor with expertise in protocol development and co-ordination of RCTs based at LSHTM. Will work closely with Diana Elbourne at the DCC and will provide expertise on trial co-ordination and data management.

6. *Marcus Richards*: senior neuropsychologist based at University College London.

7. *Graham Holder*: Director of Electrophysiology at Moorfields Eye Hospital.

8. *Louise Letley*: Senior Nurse Manager for the MRC GPRF with six years experience of co-ordinating nurse managed research projects in primary care. Has overall responsibility for the research nurses involved in the study and for the management of quality control within the practices.

### Trial Steering Committee

The overall scientific aspects of the project will be managed by a Steering Committee. The Steering Committee will include expert independent advisors Professor Sir John Grimley Evans (Chair), Professor Martin Prince, Professor Alan Bird, Dr. Madge Vickers (co-opted onto Steering Committee as independent member from January 2005), two members of the public Mrs. Ursula Shine and Mrs. Yvonne Davidson, the principal applicants, and project staff *ex-officio*. Members of the public are defined here as: patients and potential patients; informal (unpaid) carers; people who use health and social services; members of the public who may be targeted by health promotion programmes; organisations that represent the interests of people who use health and social care services. The Steering Committee will take all executive decisions.

The responsibility of the Steering Committee is to ensure the scientific integrity and quality of the project. To achieve this, the specific responsibilities of the Steering Committee include:

- maintaining adherence to the study protocol

- approving changes to study protocol if required

- reviewing quality assurance indicators

- monitoring study recruitment and the overall study timetable

- advising, as required, on specific scientific items that may arise

- compliance with legislation

- adherence to research governance

- reporting to funders

- approving publication and dissemination strategies.

The Steering Committee will meet six monthly.

### Project Management Group (PMG)

A Project Management Group (comprising principal investigators, trial manager, senior nurse, database manager and statistician) will run the trial on a day-to-day basis to ensure the smooth operation of the project. Regular review meetings will be held with other members of the team as appropriate.

The responsibilities of the PMG include:

- establishing and monitoring recruitment of participating centres and participants

- distributing and supplying data collection forms and other appropriate documentation for the trial

- distributing and supplying dietary supplements to trial practices

- data collection and management

- data entry and cleaning

- data analysis

- organising and servicing the Data Management Committee

- organising and servicing the Trial Steering Committee.

### Data Monitoring Committee

An independent Data Monitoring Committee (DMC) will be established. The membership will comprise Professor Tom Sanders (Chair), Professor Graham Dunn (senior statistician) and Dr. Gill Livingston (clinician). The role of the DMC will be firstly to check on safety by random allocation. Although this intervention is not expected to lead to adverse consequences, it is important to guard against this eventuality. If necessary, this could lead to stopping the trial before knowing the primary outcome. Secondly the DMC will also look at on-going compliance data by random allocation to check that a difference in exposure is occurring. If a problem were to be detected, this could lead to a recommendation to amend aspects of the protocol. Thirdly, the DMC will consider data at trial entry by random allocation in order to be able to interpret the compliance and side effects data. The committee will meet at the start of the trial to agree terms of reference and then at the end of the recruitment period and every 6 months or as they determine.

### Medical Research Council General Practice Research Framework (GPRF)

The responsibilities of the GPRF are to select and support general practices from which the participants are to be drawn. The GPRF will train, monitor and support research nurses throughout the trial, assure recruitment and compliance to the trial protocol, and collect high quality data as detailed in the trial protocol. The trial manager, who will liaise closely with the principal investigator, and report to the GPRF senior clinical scientist, will be based at the GPRF. In order to ensure that trial participants are adequately supported and are able to adhere to the trial regimen, the GPRF will also ensure the provision of direct phone access to the trial manager who will liaise as necessary to resolve possible problems that may affect compliance and retention.

### Data Coordinating Centre

The Data Co-ordinating Centre (DCC) will be based at the LSHTM. The responsibilities of the DCC are to set-up and run systems for data entry, data verification and the checking of errors and overdue data reports in close liaison with the GPRF, and to conduct interim and final analyses for the cognitive and retinal function studies.

### Moorfields Eye Hospital

The responsibilities of the investigator at Moorfields Eye Hospital are to carry out retinal examinations at baseline and 24-months of the sample agreeing to take part in the retinal function study, co-ordinate with the GPRF and DCC to ensure that participants attend as necessary, interpret primary data and forward cleaned data to the DCC for analysis.

### Publication policy

To safeguard the scientific rigour of the trial, data from this study will not be presented in public or submitted for publication without requesting comments and receiving agreement from the Trial Steering Committee. The primary results of the trial will be published with authorship in relation to specific participation in the study, with the name order to be presented by the PI for consideration by the Trial Steering Committee. Suggested revisions in order of authors should meet with the approval of the PI. Publications in specific areas of the study or on methodological aspects can be led by co-investigators in their area of expertise subject to approval by the Trial Steering Committee and the PI. The requirements for authorship will follow recommended practice in journal guidelines.

### Confidentiality

Participants will be identified by their trial number to ensure confidentiality. However, as the participants in the trial will be followed for 24 months following randomisation, it is essential that the teams at the GPRF have the names and addresses of the trial participants recorded on the data collection forms in addition to the allocated trial number. Stringent precautions will be taken to ensure confidentiality of names and addresses at the GPRF. The investigators and local coordinators will ensure conservation of records in areas to which access is restricted.

### Longer term follow-up

Further follow-up may be the subject of a separate protocol. So that the study organisers do not lose contact with patients should they move address and also to follow up on health status, participants are being asked to give their agreement for their names and NHS numbers to be sent to the NHS Central Register.

## Conclusion

The number of individuals with age-related cognitive impairment is rising worldwide, and public health interventions aimed at slowing this rise are urgently needed. There is a growing body of mechanistic and epidemiologic evidence to support the proposal that n-3 LCPs may reduce the risk of age-related loss of cognitive function in older people. The current trial is designed to test the hypothesis that an increased dietary intake of n-3 LCPs will have a positive effect on cognitive performance in older people in the UK. This will be the first trial of its kind aiming to slow the decline of neuronal function in older people by increasing daily dietary intake of n-3 LCPs. The link between cognitive ability and quality of life among older people suggests that this novel line of research may have considerable public health importance.

## Abbreviations

DCC – Data Co-ordinating Centre

DHA – docosahexaenoic acid

DMC – Data Monitoring Committee

EPA – eicosapentaenoic acid

ERG – electroretinogram

GHQ-30 – 30-item General Health Questionnaire

GPRF – General Practice Research Framework

GRAS – Generally recognised as safe

MI – Myocardial infarction

MMSE – Mini Mental State Examination

n-3 LCPs – n-3 long-chain polyunsaturated fatty acids

PMG – Project Management Group

## Competing interests

The author(s) declare that they have no competing interests.

## Authors' contributions

AD and RU conceived the study. AD, DE, AF, GH, MR, AT, RU and MV were applicants for the funding. All authors were involved in designing the study and drafting the protocol. FH and MR designed the cognitive testing booklet. GH designed the electrophysiological testing protocol. All authors read and approved the final protocol.
